# Mapping the Evidence on Midline Catheter Tip Location, Confirmation Methods and Associated Characteristics: A Scoping Review

**DOI:** 10.1002/nop2.70876

**Published:** 2026-09-30

**Authors:** Jieyu Wang, Si Deng, Hui Hu, Ya Feng, Xiaoling Dong, Jing Zhang, Jiao Yang, Li Geng, Yilan Liu, Yaling Wang

**Affiliations:** ^1^ Department of Neurology, Union Hospital, Tongji Medical College Huazhong University of Science and Technology Wuhan Hubei P. R. China; ^2^ Department of Rheumatology, Union Hospital, Tongji Medical College Huazhong University of Science and Technology Wuhan Hubei P. R. China; ^3^ Department of Nursing, Union Hospital, Tongji Medical College Huazhong University of Science and Technology Wuhan Hubei P. R. China

**Keywords:** catheterization, catheter‐related infections, peripheral, radiography, scoping review, ultrasonography, venous thrombosis

## Abstract

**Background:**

Midline catheters (MCs) are increasingly utilized as an alternative to peripheral and central venous catheters. However, the lack of standardized protocols for tip location and confirmation methods remains a significant barrier to patient safety and clinical decision‐making.

**Aim:**

This scoping review aimed to systematically map the current evidence and evolving clinical practices regarding MC tip placement, specifically exploring tip location standards, confirmation methods and associated clinical characteristics.

**Design:**

Scoping review.

**Review Methods:**

The scoping review was conducted following the Arksey and O'Malley framework and PRISMA‐ScR reporting guidelines. Two independent reviewers conducted screening and data extraction, with descriptive methods used for synthesis.

**Data Sources:**

Eleven databases (platforms) were searched from inception to 31 August 2026: the Cochrane Library, PubMed, EMBASE, Web of Science, Scopus, ProQuest, CINAHL, CNKI, Wanfang Data, SinoMed and VIP databases.

**Results:**

Out of 762 initial records, 41 publications met eligibility criteria. North American guidelines strictly restrict MC tips to the peripheral axilla, whereas European consensus advocates for deeper placement into the thoracic axillary or subclavian veins. Tip confirmation practices are heterogeneous. Methods range from external anatomical measurement to ultrasound guidance and post‐procedural radiography. A significant limitation identified is the reduced reliability of ultrasound for tip visualization in patients with morbid obesity, often necessitating alternative confirmation or resulting in indeterminate placement. The quality and comparability of outcome data across studies were limited by inconsistent tip location definitions, heterogeneous patient populations and variable follow‐up durations.

**Conclusion:**

This review highlights substantial heterogeneity in clinical practice and guideline recommendations. The association between tip position and catheter‐related complications is multifactorial. This scoping review identifies that current evidence is inadequate to support a clear preference for any single midline catheter tip‐positioning strategy. These findings set an agenda for future research to inform standardized, evidence‐based midline catheter practice guidelines.

**Implications for the Profession:**

Clinical nurses and vascular access specialists should be aware of divergent international guideline suggestions for midline catheter tip positioning. Consensus‐based definitions for implantation depth and target veins, together with consistent anatomical terminology, are needed to support cross‐study comparison. Optimized multimodal tip‐confirmation workflows may reduce unnecessary X‐ray use and improve safety, supported by targeted education and interprofessional collaboration. Future prospective cohort studies and comparative trials are needed to evaluate the relationship between tip location, confirmation strategies and patient‐important clinical outcomes.

**Impact:**

This scoping review synthesizes existing international literature on midline catheter tip‐related practices and exposes inconsistencies across guideline documents. It draws attention to key unresolved clinical questions and provides clear directions for subsequent primary research. The findings may support the development of future consensus statements and help reduce practice variation in midline catheter care.

**Reporting Method:**

PRISMA‐ScR.

**Patient or Public Contribution:**

No patient or public contribution.

**Review Protocol Registration:**

https://osf.io/fn8vz/overview.

## 1 Introduction

Vascular access devices (VADs) are indispensable components of modern medical practice, with billions of catheters inserted globally each year to support medication delivery, fluid therapy, blood sampling and hemodynamic monitoring (Nickel et al. 2024). Among these, midline catheters (MCs) have emerged as a valuable alternative, balancing the safety profile of peripheral catheters with the extended dwell time of central lines (Wen et al. 2025). Specifically, a midline catheter (MC) was defined as a peripheral catheter inserted into the basilic, cephalic, or brachial vein of the upper arm, with the terminal tip located at the level of the axilla in children and adults according to the 2024 Infusion Therapy Standards of Practice (Nickel et al. 2024). This unique positioning offers significant advantages: MCs provide extended dwell times and lower mechanical complication rates compared to short peripheral intravenous catheters (PIVs) (Rivas et al. 2025; Sakakida et al. 2025), while circumventing the central venous cannulation and technically less demanding to place, and avoiding the associated complications of peripherally inserted central catheters (PICCs) and other central venous catheters (Chopra et al. 2019; Long et al. 2025). Consequently, with the increasing demand for intermediate‐duration intravenous therapy (1–4 weeks), such as prolonged antibiotic therapy and chemotherapy, and the global shift towards outpatient and home‐based care models, the clinical utilization of MCs has grown substantially in recent years (Meto et al. 2025; Paje et al. 2025; Sakakida et al. 2025).

Despite robust evidence linking precise tip location to improved outcomes, clinical practice remains fragmented due to inconsistent guidelines, variable training and lack of global standardization. For instance, the Infusion Nurses Society (INS) 2024 Practice Standards define the ideal MC tip location at the level of the axillary vein (Nickel et al. 2024). In contrast, the Global Vascular Access Network (GloVANet), in collaboration with the World Congress of Vascular Access (WoCoVA) indicated that the tip of the MC may be located in the thoracic tract of the axillary vein or even extend into the subclavian vein (van Rens et al. 2025). Such definitional discrepancies not only create operational confusion for frontline clinicians but also propagate into clinical workflows, where institutions adopt disparate protocols based on local consensus rather than unified evidence, ultimately leading to unwarranted variations in patient risk. Furthermore, this lack of uniformity severely impedes comparative analyses across clinical studies.

Similarly, MC tip confirmation methods remain controversial. Compounding the issue of anatomical placement is the ongoing controversy surrounding MC tip confirmation methods. Conventionally, chest radiography (X‐ray) has served as the ‘gold standard’ for confirming central venous catheter tips; however, its necessity for MCs—and whether point‐of‐care ultrasound (POCUS) can safely and effectively replace it—remains highly debated (Sou et al. 2017). Point‐of‐care ultrasound (POCUS) has emerged as a powerful, non‐invasive alternative, offering real‐time visualization at the bedside, although its various techniques and validation against traditional methods are still under investigation. Similarly, while intracavitary electrocardiographic (IC‐ECG) guidance is now widely adopted for PICC tip positioning, its diagnostic value and feasibility for MC placement have yet to be definitively established (Gullo and Qanadli 2022). Resolving these controversies regarding accurate placement and confirmation is critical to minimizing catheter‐related complications (Fu et al. 2023).

Several recent reviews have quantitatively evaluated the relationship between MC tip location and catheter‐related complications. Zhang et al. conducted a systematic review and conventional meta‐analysis and found that, compared with axillary vein placement, subclavian vein placement was associated with a lower overall complication rate and a reduced risk of catheter‐related thrombosis, whereas differences in dwell time, phlebitis, occlusion, infiltration, pain, and dislodgement were not statistically significant (Zhang et al. 2024). Sheng et al. subsequently included nine studies and reported that MC tips located in the subclavian vein were associated with lower rates of overall catheter‐related complications, phlebitis, bleeding, infiltration, occlusion, dislodgement and catheter‐related thrombosis, as well as longer dwell time, compared with axillary vein placement (Sheng et al. 2025). More recently, Yang et al. used Bayesian network meta‐analysis to compare multiple anatomical tip locations and reported that subclavian vein placement ranked favourably for reducing thrombosis, occlusion, phlebitis and overall complications (Yang et al. 2025); however, the analysis also highlighted uncertainty related to small sample sizes, wide credible intervals for some comparisons and heterogeneous tip‐confirmation methods.

These reviews provide important quantitative evidence, but their scope is necessarily focused on pooled complication estimates. Several questions that directly affect clinical standardization remain insufficiently mapped. First, anatomical terminology varies substantially across studies and guidelines, with terms such as distal axillary vein, proximal axillary vein, thoracic axillary vein, axillo‐subclavian junction, subclavian vein, and brachiocephalic vein used inconsistently. Second, existing meta‐analyses do not comprehensively map how tip location was determined or confirmed, including surface measurement, real‐time ultrasound, microbubble or saline‐enhanced ultrasound, radiography and hybrid approaches. Third, patient‐level and device‐level characteristics that may influence tip accuracy and complications—such as catheter‐to‐vein ratio, obesity, vein diameter, catheter length, insertion site, infusion properties and follow‐up duration—remain inconsistently reported. Fourth, because previous reviews primarily synthesized outcomes, they provide limited information on implementation issues, clinical workflows and evidence gaps relevant to future protocol development.

Therefore, the purpose of this scoping review is not to duplicate prior meta‐analyses or to generate a new pooled effect estimate. Instead, it aims to map the breadth and nature of evidence on MC tip location, confirmation methods and associated clinical characteristics; to compare how different studies and guidelines conceptualize terminal tip location; to summarize how existing findings align or conflict with recent quantitative reviews; and to identify methodological gaps requiring future prospective research.

### 1.1 Aim

The overarching objective of this scoping review is to map and synthesize the existing evidence concerning the tip location of MCs. Specifically, this review aims to answer the following research questions:

1. How does the existing literature define the ideal tip location for MCs?
2. What methods are used in clinical practice to confirm MC tip location?
3. What factors influence the accuracy of MC tip positioning?
4. What clinical complications and outcomes have been investigated or described in the literature in relation to MC tip position?

## 2 Methods

### 2.1 Design

Scoping review methodology is particularly appropriate for emerging research areas with immature evidence bases and broad research questions, enabling effective mapping of the scope and distribution of existing evidence, identification of research gaps and direction‐setting for future inquiry (Peters et al. 2021). The protocol of this scoping review was registered and published on OSF (https://osf.io/fn8vz/overview). This study followed the scoping review framework by Arksey and O'Malley (Arksey and O'Malley 2005), enhanced with specific operational recommendations from Levac et al. (Levac et al. 2010) concerning the iterative team‐based processes for study selection and data charting (Stages 3 and 4). Reporting of this review adheres to the Preferred Reporting Items for Systematic reviews and Meta‐Analyses extension for Scoping Reviews (PRISMA‐ScR) checklist (Tricco et al. 2018).

### 2.2 Search Methods

Systematic searches were performed across the Cochrane Library, PubMed, EMBASE, Web of Science, Scopus, ProQuest, CINAHL, CNKI, Wanfang Data, SinoMed, VIP databases from inception to August 31st, 2026. The search strategies were developed for each database using database‐specific controlled vocabulary, where applicable, together with free‐text keywords. Controlled vocabulary was used for concepts for which an appropriate subject heading was available. However, no single controlled‐vocabulary term adequately captured the concepts of ‘midline catheter’ and ‘catheter tip’ across all databases. These concepts were therefore searched primarily using free‐text terms, including synonyms, spelling variants, truncation, phrase searching and related terminology. Key search terms included:

‘midline catheter*’ OR ‘mid‐line catheter*’ OR ‘midline venous catheter*’ OR ‘midline peripheral catheter*’ OR ‘medium‐term intravenous access*’ OR ‘medium‐long catheter*’ OR ‘midlength catheter*’ OR ‘mid‐length catheter*’ OR ‘medium and long catheter*’ OR ‘midclavicular catheter*’ OR ‘long peripheral catheter*’ OR ‘long peripheral cannula’ OR ‘extended dwell catheter’; ‘tip’ OR ‘position’ OR ‘positioning’ OR ‘location’ OR ‘subclavian’ OR ‘axillary’ OR ‘brachiocephalic’ OR ‘orientation’. Search strategies were adapted for each database according to its specific syntax requirements. The Search strategies used in data sources were shown in Table S1. To identify relevant reviews, all articles that either were referenced by or reference to the included reviews, were scrutinized for inclusion.

### 2.3 Eligibility Criteria

Studies were included if they included the following information: (1) Patients (≥ 18 years old) receiving midline catheters for venous access; (2) Studies must report on the tip location of midline catheters, methods used to confirm tip location and/or characteristics of the catheter tip. In this scoping review, a midline catheter is ≥ 15 cm. Studies focusing solely on peripherally inserted central catheters (PICCs) or standard peripheral IVs will be excluded unless they specifically discuss midline catheters or provide comparative data relevant to midline catheters; (3) The review was limited to English and Chinese publications due to the research team's language proficiencies and the predominant representation of these languages in the biomedical literature on this topic. Studies were excluded if: (1) studies exclusively focusing on shorter MCs (< 15 cm), usually called ‘mini‐midlines’ or ‘short midlines’ or ‘long peripheral cannulas’, (2) review, study protocols, editorials, opinion pieces, book chapters without original research data, letters to the editor (unless they report novel findings), (3) studies published in languages other than those specified in the language inclusion criteria, (4) grey literature not readily accessible or verifiable.

### 2.4 Study Selection

All retrieved records were imported into Zotero (V 7.0.30) for reference management. After automated deduplication, two independent reviewers (S.D. and J.Y.W.) screened all records in two rounds using predefined eligibility criteria: initial screening by title and abstract, followed by full‐text review. Disagreements were resolved through discussion and consensus, with arbitration by a third reviewer when necessary.

### 2.5 Data Extraction

A pre‐designed data extraction form was used to extract the following information: first author and publication year, country/region, title, study design, population, sample size, MC characteristics (material, length, type, dwell time), tip position confirmation method, type and incidence of complications. Extraction was performed in duplicate. Due to the limitations of resources and time, no requests for unreported results were submitted to research authors.

### 2.6 Data Synthesis and Analysis

A narrative synthesis approach was used to analyse and integrate extracted data. Included literature was categorized into the following thematic areas: tip position definitions and classification standards; tip position confirmation methods; factors influencing tip position accuracy; and relationships between tip position and complications. Descriptive statistics were used to report characteristics of included studies.

## 3 Results

### 3.1 Study Selection

Database searches yielded 762 records. After deduplication, 398 records were removed, leaving 364 unique records for screening. Title and abstract screening excluded 231 records, leaving 133 for full‐text review. After full‐text assessment, 41 publications met eligibility criteria. Reasons for exclusion were: not midline catheter (*n* = 3, predominantly mini midline catheters), non‐tip positioning (*n* = 34), irrelevant to the scoping review topic (*n* = 22), incomplete full text (*n* = 24; 21 conference abstracts and 3 study protocols), wrong language (*n* = 1), wrong study population (*n* = 1) and full text not retrievable (*n* = 7). Among the included publications, two articles (Zhao et al. 2022, 2023) were identified as companion publications originating from the same clinical trial. Additionally, one journal article (Che et al. 2026) and a Master's thesis (Che 2025) were approved by the same ethical review committee with the same approval ID and were also identified as companion publications. To prevent double‐counting in sample‐size aggregation and primary study counts, these overlapping publications were consolidated, representing 39 unique studies. All 41 individual publications were retained and charted separately in the evidence‐mapping tables to capture their distinct outcome endpoints.

The PRISMA flowchart is shown in Figure 1.

**FIGURE 1:**
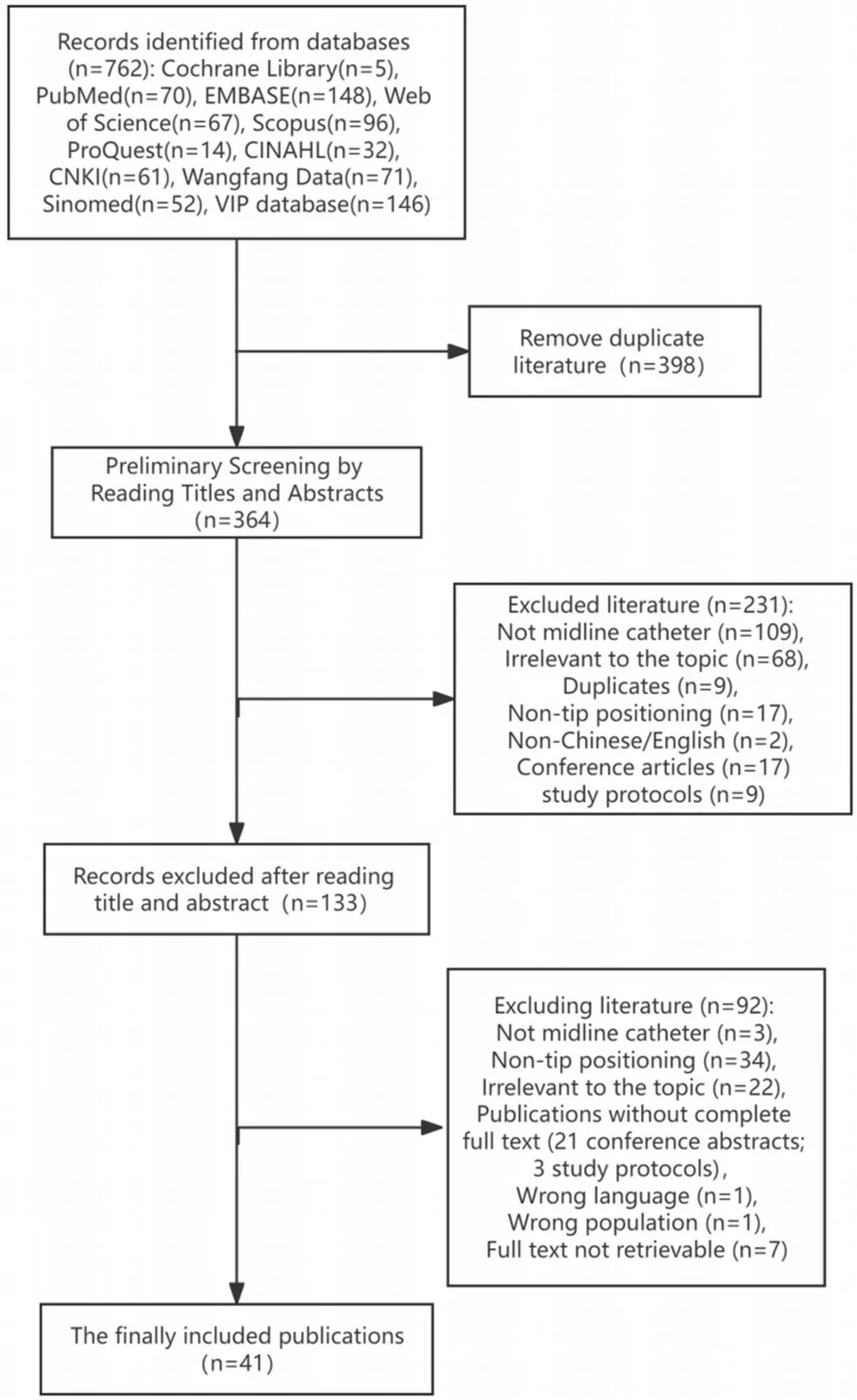
PRISMA flowchart of study selection.

### 3.2 Characteristics of Included Studies

Studies publication year distribution: 39 studies were published between 2015 and 2026, with 71.79% (28/39) published after 2020, indicating substantially increased research activity in recent years. Geographic distribution: Included studies originated only from 4 countries, predominantly China (*n* = 30, 76.92%), Italy (*n* = 7, 17.95%), the United States (*n* = 1, 2.56%) and India (*n* = 1, 2.56%), highlighting significant geographic bias in this literature. Of these two companion publications, Che et al. (2026) was designed as a non‐randomized controlled trial (NRCT), whereas Che (2025) was a randomized controlled trial (RCT). Study design distribution of the included publications: randomized controlled trials (RCTs) (*n* = 11, 26.83%), non‐randomized controlled trials (NRCTs) (*n* = 2, 4.88%), cohort studies (*n* = 15, 36.59%), descriptive studies (*n* = 9, 21.95%), self‐controlled studies (*n* = 1, 2.44%), historical control studies (*n* = 1, 2.44%) and case series (*n* = 1, 2.44%). Sample size range: Sample sizes in primary studies ranged from 12 to 2307 participants or catheter‐placement episodes. Detailed characteristics of included publications were shown in Table S2.

### 3.3 Tip Location Definitions and Classification Standards

Midline catheters (MCs) occupy a distinct anatomical and clinical niche within the vascular access device (VAD) spectrum, positioned between peripheral intravenous catheters (PIVCs) and centrally inserted central catheters (CICCs). Despite the physiological advantages of deeper placement, definitional consensus regarding the ideal MC tip location remains notably inconsistent across guidelines globally. The scoping review identified a significant evolution in recommendations, reflecting both the accumulation of clinical evidence and a divergence in regional clinical philosophies shown in Table 1.

**TABLE 1:** Summary recommendations for MC tip location.

Guidelines	Year	Recommended tip location	Region
MAGIC	2015	In the basilic or cephalic vein	North America
INS	2016	At or near the level of the axilla	North America
INS	2021	At the level of the axilla	North America
RNAO	2021	Near or at the level of the axilla and distal to the shoulder	Canada
ERPIUP	2023	Thoracic tract of the axillary vein or subclavian vein	Europe
INS	2024	At level of axilla	North America
GloVANet‐WoCoVA	2025	Thoracic tract of the axillary vein or subclavian vein	International

#### 3.3.1 North American Perspective

Historically, definitions strictly limited MC tips to the peripheral vasculature. The Infusion Nurses Society (INS) 2016 Practice Standards recommended the tip be located ‘at or near the level of the axilla’ (Gorski 2017). This was explicitly maintained in the INS 2021 standards (Gorski et al. 2021) and reiterated in the 2024 INS guidelines, which mandate positioning the MC tip at the level of the axilla, strictly outside the central circulation (Nickel et al. 2024). Similarly, the 2015 Michigan Appropriateness Guide for Intravenous Catheters (MAGIC) defined MCs as being inserted 8–20 cm with the tip terminating in the basilic or cephalic vein (Chopra et al. 2015), and the Registered Nurses' Association of Ontario (RNAO) 2021 recommends the tip reside near or at the level of the axilla and distal to the shoulder (Registered Nurses' Association of Ontario 2021).

#### 3.3.2 European and Emerging Perspectives

Conversely, European consensus has increasingly advocated for deeper placement to minimize complications. The 2023 European Recommendations for Proper Indication and Use of Peripheral venous access (ERPIUP) declared that, without trimming, the MC tip can be safely located in the thoracic tract of the axillary vein or in the SV (Pittiruti et al. 2023). This paradigm shift is further solidified in the 2025 GloVANet–WoCoVA, which explicitly states that the tip of the MC should be located in the thoracic tract of the axillary vein or even within the subclavian vein (van Rens et al. 2025).

#### 3.3.3 Other Regions

Demonstrating further heterogeneity, the 2020 Chinese Expert Consensus on MC Clinical Application (Nursing Branch of the China Research Hospital Association) did not recommend a universally specific anatomical position for the MC tip, highlighting the need for individualized assessment (Project Team of Nursing Branch of China Research Hospital Association 2020).

### 3.4 Tip Position Confirmation Methods

Tip confirmation practices for MCs are substantially less standardized than for those for centrally inserted central catheters (CICCs) or peripherally inserted central catheters (PICCs). This heterogeneity reflects both the historically assumed lower‐risk nature of non‐central placement and resource‐related clinical pragmatism. The methods identified for MC tip confirmation fall broadly into three categories: external body measurement, ultrasound technology and plain chest radiography. Study about Tip Position Confirmation Methods were shown in Table 2.

**TABLE 2:** Publications about tip position confirmation methods.

Author(s) (year)	Catheter type	Catheter tip position (*n*)	Catheter dwell time (days)^a^	External body measurement	Ultrasound technology	Plain chest radiography
Yu et al. (2026)	4F, 30 cm MC from Subekang Company	Subclavian vein (43)	25.3 ± 4.2	—	Examine the tip of the catheter with ultrasound	—
Chen et al. (2026)	Subekang 4 Fr, 30 cm three‐way valve single‐lumen silicone catheter	Middle section of the subclavian vein (80)	25.68 ± 0.54	—	Confirmed by ultrasonic positioning	—
Che et al. (2026)	—	Thoracic segment of the axillary vein (96); Subclavian vein (95)	—	Thoracic segment of the axillary vein: the distance from the pre‐puncture site to the midline of the ipsilateral clavicle minus 3 to 4 cm; Subclavian vein: from the pre‐puncture site to the ipsilateral sternoclavicular joint minus 1 to 2 cm.	Once the catheter is advanced to the target site, ultrasound is applied to confirm tip location. The probe is positioned perpendicular to the vessel short axis below the axillary horizontal line, then rotated parallel to the vessel long axis after crossing the axillary plane. Following identification of the axillary vein, the probe is advanced towards the sternoclavicular joint. At the midclavicular line, the catheter tip (visualized as double horizontal echoes on ultrasound) is positioned 3–4 cm from this landmark. As the clavicle obscures visualization of the subclavian vein, the final tip position is determined by anatomical estimation and external measurement: 1–2 cm proximal to the sternoclavicular joint.	—
Che (2025)	35 cm Kangxin open‐tip 4 Fr single‐lumen catheter kit (Shandong Biodan Medical Devices Co. Ltd.)	3~4 cm lateral to the midline of the clavicle (119); Midline of the clavicle (119); Middle and inner third of the clavicle (119); 1~2 cm lateral to the sternoclavicular joint (119)	—	From the pre‐puncture site to 3–4 cm lateral to the midclavicular line; From the pre‐puncture site to the midclavicular line (±1 cm); From the pre‐puncture site to the medial one‐third of the clavicle (±1 cm); from the pre‐puncture site to 1–2 cm lateral to the sternoclavicular joint.	The assistant confirmed the catheter tip position under ultrasound imaging.	—
Zeng et al. (2025)	4 Fr Three‐way valve‐type medium‐length venous catheter	Subclavian vein (12)	5–12	Distance from the pre‐puncture site to the ipsilateral sternoclavicular joint.	—	—
Passaro et al. (2025)	Open‐ended, non valved polyurethane catheters (Midline, Deltamed), 25 cm long, singlelumen (4 or 5 Fr) or doublelumen (5 Fr)	Thoracic tract of the axillary vein (19); subclavian vein (12); transition between the axillary/subclavian veins or the brachial tract of the axillary vein (10)	7–10	—	Performed with compression ultrasonography (CUS), plus crosssectional/longitudinal scans with the use of colorDoppler.	—
Nie et al. (2025)	The SPECATH 4 Fr silicone catheter (Fo Shan Special Medical, China) was used, a 30 cm single‐lumen, nonhigh‐flow catheter with a fixed, one‐piece design and a proximal three‐way valve. It could not be cut or adjusted in length	Axillary vein (1124); Subclavian vein (1183)	10 (7, 15); 10 (7, 15)	Axillary vein group: from the puncture point to the ipsilateral midclavicular; Subclavian vein group:2 cm was deducted from the distance from the puncture point to the ipsilateral sternoclavicular.	—	Postcatheterisation, a chest X‐ray confirmed the tip position.
Zhu et al. (2025)	—	Subclavian vein (40); Thoracic tract of the axillary vein (40)	15.27 ± 2.18; 12.09 ± 1.94	Subclavian vein: Measure from the labeling point along the course of the axillary vein to the distance to the ipsilateral subscapular region or axilla; Thoracic tract of the Axillary vein: Measure from the labeling point along the course of the axillary vein to the ipsilateral sternoclavicular joint, then subtract 2 cm.	—	—
Yu et al. (2025)	—	Subclavian vein (100)	A month	From puncture site to the mid‐clavicular point on the same side.	When the length is 15~20 cm, the assistant places the B‐ultrasound probe at the patient's ipsilateral subclavian axillary vein thoracic segment, and the operator continues to slowly feed the catheter, and when the catheter tip reaches the axillary vein thoracic segment, the B‐ultrasound screen can observe the image of the catheter tip, and the operator continues to feed the catheter 2~3 cm after determining the catheter exposed scale to ensure that the catheter tip reaches the subclavian vein.	—
Xiong and Zhang (2025)	—	Axillary vein (15); Subclavian vein (15)	—	—	—	—
Schechter et al. (2024)	Uncoated 20‐cm Bard PowerMidline (CR Bard Inc.)	Arm vein (41); Axillary vein (18)	12 (IQR = 11); 8 (IQR = 10)	—	An upper extremity duplex examination performed by the vascular laboratory within 90 days of midline catheter placement.	The lateral border of the scapula on chest X‐ray is the anatomic boundary for the axillary vein; therefore, any midline catheter tip proximal to this border on chest X‐ray was considered to be in the axillary vein and any tip distal to it was considered to be in an arm vein.
Fabiani et al. (2024)	18 G (single‐lumen), 4 Fr (1.32 mm), 20 cm; 19/21 G (duallumen), 4 Fr (1.40 mm), 20 cm; 18/18 G (dual‐lumen), 5 Fr (1.67 mm), 20 cm.	Catheter‐to‐vein ratio > 33% at the tip level (1); Catheter‐to‐vein ratio ≤ 33% at the tip level (130)	26.6 ± 23.8	—	Final ultrasound assessment of the catheter position up to the tip location.	—
Jiang et al. (2024)	Split‐type three‐way valved silicone catheters of the same brand and batch number were used, with an overall length of 30 cm.	Axillary vein (91); Subclavian vein (93)	—	Axillary vein group: from the puncture site to the midclavicular line, then minus 3 to 4 cm from this measurement; Subclavian vein group: from the pre‐puncture site to the ipsilateral sternoclavicular joint, with an additional subtraction of 2 cm.	—	Chest X‐ray.
Zhao et al. (2023)	The same brand and batch number of silicone‐valved catheters, with a total length of 30 cm. Catheters could be trimmed to ensure a consistent length of the external catheter	Subclavian vein (109); Axillary vein of the chest wall (89)；Distal to the axillary vein (83)	15.95 ± 10.63; 17.10 ± 15.72; 13.88 ± 10.76	Subclavian vein: 2 cm was subtracted from the pre‐puncture point to the ipsilateral sternoclavicular joint to calculate the effective catheter preplacement length；Axillary vein of the chest wall: subtracting 3–4 cm from the distance between the puncture point and ipsilateral midclavicular line；Distal to the axillary vein: the distance from the pre‐puncture point to the ipsilateral sub shoulder or axilla was measured.	An intravenous therapy (IV) specialist nurse confirmed catheter tip positioning by ultrasound during catheterization, and the position of the catheter tip was adjusted according to ultrasound imaging.	X‐ray imaging was performed after catheterization to confirm the position of the catheter tip.
Marche et al. (2023)	Inserted power injectable, non‐valved, polyurethane, single lumen MCs of two different brands (Medcomp and Vygon); catheter length ranged between 20 and 25 cm, and the lumen was approximately 16–17G for 5 Fr catheters or 18G for 4 Fr catheters.	Thoracic tract of the axillary vein (69)	—	—	Assessing tip location by ultrasound.	—
Gidaro et al. (2023)	MedComp 4 F × 20 cm, single lumen, power injectable	—	—	—	Contrast enhanced ultrasound.	Chest X‐Ray.
Fu et al. (2023)	Per‐Q‐Cath 4 Fr catheters from Bard Access Systems Inc	Distance between the catheter tip and the clavicle was > 3 cm (53); Distance between the catheter tip and the clavicle was ≤ 3 cm (42)	18.5 (11, 31); 29 (14, 77)	Either from the pre‐puncture site along the vein to the axillary level or from the pre‐puncture site along the vein to the ipsilateral clavicular midline minus 2 cm.	The position of the catheter tip was localized to where the shadow in the ultrasound of the catheter disappeared. Results were evaluated and confirmed by at least two qualified ultrasound technicians.	—
Fabiani et al. (2023)	Polyurethane power‐injectable 4 5 Fr, 18 G, 20 cm, single or dual‐lumen MCs (PowerMidlineTM, Becton Dickinson, Salt Lake City, USA).	Catheter‐to‐vein ratio ≤ 45% at the tip level (126)	25.7 ± 20.4	—	Ultrasound	—
Yu et al. (2023)	Shubekang Company's 3 Fr modified medium‐long catheter (35 cm)	Subclavian vein (85); thoracic tract of the Axillary vein (20); Brachiocephalic vein (15)	19.66 ± 15.99; 11.26 ± 6.18; 20.38 ± 15.69	Measure the pre‐placed catheter length without explanation.	—	Chest X‐ray to locate the tip of the catheter.
Xiong et al. (2023)	Shubekang Tip 3‐way Valve‐Type 4 Fr Single‐Lumen Catheter, with a catheter diameter of 1.34 mm and a total length of 30 cm	Thoracic tract of the Axillary vein (105)	13.00 ± 7.00	The distance from the puncture point to the observation point of the axillary vein in the thoracic segment on the skin.	Detect with a Site Rite 5 ultrasound machine to confirm that the catheter tip reaches the reservation.	—
Mao (2023)	—	Anterior axillary line (75); Midclavicular line (75)	—	—	—	—
Jing (2023)	Foshan Special Medical Catheter Co. Ltd. produces disposable peripheral puncture central venous catheter, model PB‐3 Fr‐1‐A, specification 30 cm	Axillary vein (100)	—	—	Ultrasound	—
Chen et al. (2023)	Shubecon modified three‐way valve medium‐length catheter (single‐use PICC) with catheter model 4 Fr, length of 30 cm, flow rate of 12 mL/min	Subclavian vein (80)	—	—	—	—
Zeng et al. (2023)	Silicone material 4 Fr three‐way valve catheter, available in two total lengths of 30 cm and 35 cm. Select the appropriate model based on the implantation length and perform self‐cutting.	Subclavian vein (216)	—	The distance from the prepuncture point along the vein to the ipsilateral sternoclavicular joint minus 2 cm.	Ultrasound	—
Thiyagarajanand and Ravindran (2022)	A single lumen conventional central venous catheter (5 Fr, 20 cm)	Axillary vein (72)	12 (6, 20)	—	Ultrasound	—
Zhao et al. (2022)	Split‐type three‐way valve silicone catheter with a total length of 30 cm, allowing for self‐trimming of the catheter	Subclavian vein (141); The thoracic tract of the axillary vein (123); distal to axillary vein (120)	12 (8, 19.75); 12 (8, 21); 10 (6, 17.75)	Subclavian vein: distance from the pre‐puncture site to the ipsilateral sternoclavicular joint minus 2 cm. The thoracic tract of the axillary vein: distance from the puncture site to the ipsilateral midclavicular line minus 3 to 4 cm. Distally to axillary vein: distance from the pre‐puncture site to the ipsilateral deltoid‐thoracodorsal intermuscular groove (not exceeding the intermuscular groove and not reaching the axilla). If the puncture was performed at the cephalic vein, measure the distance from the pre‐puncture site to the ipsilateral subscapular region or axilla.	Ultrasound	Radiography.
Yang et al. (2022)	3 Fr single‐lumen central venous catheterization kit via peripheral vein (Shubeikang brand), with a medical‐grade silicone catheter measuring 30 cm in length and featuring a three‐way valve at the distal end opening.	Thoracic segment of the axillary vein or Subclavian vein (100)	43.5 ± 5.56	The length of the tube is placed from the puncture point to the midpoint of the clavicle.	Colour Doppler Ultrasound before preparing for extubation.	—
Bai et al. (2022)	BD Company's Per‐Q‐Cath safety‐type preconnected PICC medium‐length catheter, model 4 Fr single‐lumen catheter, with front‐trimmed design	Thoracic segment of axillary vein (40); Subclavian vein (46)	—	Thoracic segment of axillary vein: measurements were taken from the pre‐puncture site along the course of the axillary vein to the midclavicular line. Subclavian vein: measurements were taken from the pre‐puncture site along the course of the axillary vein to the sternoclavicular joint on the puncture side minus 2 cm.	Axillary vein thoracic segment: Ultrasound exploration of the head vein merges into the axillary vein, which is the axillary vein thoracic segment. Subclavian vein: Ultrasound exploration of the external cervical vein flowing into the subclavian vein.	—
Jin et al. (2021)	The catheter type is selected according to the optimal ratio of catheter diameter to vessel diameter of 45%	Axillary vein (135), subclavian vein (774)	7.59% > 28 days	The distance from the pre‐puncture point to the intersection of the inner and middle 1/3 of the inferior clavian margin.	—	—
Chen (2021)	Shubekang improved medium‐length catheter, catheter material is high‐grade medical silicone material, three‐way valve structure, catheter specification is 4 Fr, length 30 cm	Axillary vein (147); Subclavian vein (55)	14.65 ± 3.72	The length from the puncture site to the midpoint of the clavicle	—	—
Elli et al. (2020)	20 cm 4 Fr and 5 Fr MCs (CT Midline, MedComp); 25 cm, 4 Fr polyurethane MCs (Midline LifeCath, Vygon); 20 cm, 4 Fr and 5 Fr MCs (CT Midline, MedComp)	Axillary vein (458)	—	—	Ultrasound	—
Yang et al. (2020)	Three‐way valvular medium‐long catheter, catheter leghth 25 cm or 35 cm	Catheter leghth 25 cm (101); catheter leghth 35 cm (167)	16.4 ± 7.8 (27.3 ± 26.2)	Measure the length from the puncture point to the right sternoclavicular joint. The 25 cm tube measures 22 cm in length, and the 35 cm tube measures 32 cm in length.	If the length of the catheter reaches the right clavicular joint, use B‐ultrasound to locate and confirm whether the catheter has reached the innominate vein or the superior vena cava.	If the length of the catheter reaches the right clavicular joint, use chest X‐ray to locate and confirm whether the catheter has reached the innominate vein or the superior vena cava.
Wu et al. (2020)	BD Company Per‐Q‐Cath catheter	Subclavian vein (83); axillary vein (20); Brachiocephalic vein (8)	19.66 ± 15.99; 11.26 ± 6.18; 20.38 ± 15.69	When the catheter tip is located at the axillary vein or subclavian vein, the catheter length is determined as the distance from the puncture site to the ipsilateral anterior axillary line plus an additional 4–5 cm for intracorporeal catheterization. When the catheter tip is positioned at the brachiocephalic vein, the catheter length is calculated as the distance from the puncture site to the ipsilateral anterior axillary line plus an additional 8 cm. The catheter must retain a remaining length of 3 cm outside the body.	—	X‐ray.
Wang et al. (2020)	4F*40 cm, Intracatheter diameter 1.33 mm	Axillary vein (126)	13.89 ± 2.21	Measure the prepuncture point to the midclavicular line minus 2 cm.	Use the B‐ultrasound machine to view the internal jugular vein and subclavian vein on the side of the catheter to rule out catheter ectopia.	—
Lang et al. (2020)	Standard 4F opening, with a non‐trimmed three‐valve catheter tip	Axillary vein (60); Subclavian vein (60)	—	Pre‐assessed length without explanation.	B‐ultrasound confirmed that the catheter was in the blood vessel.	—
Gu et al. (2020)	Shu Beikang Modified Catheter	Axillary vein (67); Subclavian vein (67)	7–34; 3–71	Distance from puncture site to midclavicular line.	—	—
Liu et al. (2019)	Guangdong Shubeikang Company, length: 35 cm	Brachiocephalic vein (80)	154.58 ± 3.32	35 cm.	Use an ultrasound probe to explore the supraclavicular fossa of the neck and the ipsilateral axilla.	—
Liu and Li (2019)	—	Axillary vein (42); subclavian vein (42)	—	Subclavian vein: the distance from the pre‐puncture site to 2 cm lateral to the mid‐clavicular point on the same side. axillary vein: the distance from the pre‐puncture site to the level of the ipsilateral axilla.	Ultrasound	X‐ray.
Bai and Guo (2019)	Bard disposable medium‐length catheter puncture kit from the United States	Anterior axillary line (40); Mid‐clavicular line (40)	15.2 ± 8.6; 15.4 ± 1.1	Pre‐assessed length without explanation	—	—
Wang et al. (2016)	4 Fr single‐lumen one‐time use of a central venous catheter kit	Axillary vein (117), Upper part of the cephalic vein (64), Para axillary vein branch (4)	13.56 ± 4.52	Measure the length from the puncture point to the acromioclavicular joint	—	—
Zerla et al. (2015)	4F single‐lumen, silicone, valved tip midline catheter (Groshong midline, Bard Access Product)； 4F single‐lumen, polyurethane midline catheter (LifeCath Midline; Vygon, France)； 4F single‐lumen, polyurethane midline catheter (Medical Components Inc., Herleysville, PA)	Midline open tip (315); closed‐tip (308)	—	—	—	—

Abbreviation: —, not reported.

^a^ Catheter dwell time was reported heterogeneously across the included studies. Values are presented as reported in the original publications, including mean ± standard deviation, median with interquartile range, median with range, range only, or group‐specific dwell time.

#### 3.4.1 External Body Measurement

Estimating catheter insertion depth by measuring external body surface distances—such as the distance from the insertion site to desired final tip location—represents the most straightforward method currently used in clinical practice and is the approach most commonly adopted by bedside nurses. The literature describes several formulaic approaches based on the desired final tip location shown in Table 3.

**TABLE 3:** External measurement methods for MC tip placement.

Target vein	Measurement method (from puncture site)
Brachiocephalic vein	Distance from the pre‐puncture point to ipsilateral anterior axillary line + 8 cm
Subclavian vein	Distance from the pre‐puncture point ipsilateral sternoclavicular joint − 2 cm
Thoracic tract of axillary vein	Distance from the pre‐puncture point to ipsilateral midclavicular line (arm abducted)
	Distance from the pre‐puncture point to ipsilateral midclavicular line − 3 to 4 cm
	Distance from the pre‐puncture point to ipsilateral midclavicular line − 2 cm
Distal to axillary vein	(For basilic and brachial veins) Distance from the pre‐puncture point to the intermuscular sulcus of the ipsilateral deltoid muscle and pectoralis major muscle (ensuring the tip did not surpass this sulcus or reach the axilla)
	(For cephalic vein) Distance from the pre‐puncture point to ipsilateral shoulder/axilla

While pragmatic, these external measurements are highly susceptible to anthropometric variation and patient positioning, lacking the precision of real‐time or radiological confirmation. For instance, a study involving external measurements of catheter tip location in 125 patients reported that confirmatory ultrasound assessment showed 88.1% of the catheter tips were positioned at the expected site (Wang et al. 2020).

#### 3.4.2 Ultrasound Technology

Ultrasound‐guided insertion has emerged as a cornerstone of modern vascular access, with 26 included publications (63.41%) describing its use for MC tip confirmation. US enables real‐time visualization of venous anatomy and catheter trajectory, effectively reducing mechanical complications such as arterial puncture, hematoma and malposition.

##### 3.4.2.1 Anatomical Landmarks and Visualization

The Axillary Vein (Thoracic Segment) is defined sonographically by the point where the cephalic vein drains into it. The subclavian vein is identified at its junction with the external jugular vein (Bai et al. 2022). When using a linear probe in the infraclavicular area of adult patients, the first rib (which marks the start of the subclavian vein) is often obscured by the clavicle in a normal supine position. Therefore, the visualized vein is typically the axillary vein (Žarskus et al. 2023). Visualization of the subclavian vein is only possible in a few patients when the shoulder is forced into an elevated position, allowing the first rib to be seen and the axillary/subclavian vein transition to be identified (Annetta et al. 2026; Choi et al. 2020). If the catheter tip is not clearly visualized under ultrasound after insertion, an alternative technique involves uniform injection of normal saline and utilizing the briefly and rapidly appearing blood flow signal to assess the tip's location (Lang et al. 2020; Sakakida et al. 2025).

##### 3.4.2.2 Application in Confirmation

Fu et al. (2023) investigated an ultrasound‐guided method for precise catheter tip placement in the axillary vein, aiming for a position roughly 3 cm distal to the clavicle's periosteal shadow. Superficial implementation or deep implementation, based on the location of the MC, when the distance between the catheter tip and the clavicle was > 3, or ≤ 3 cm, respectively (Fu et al. 2023).

Yu et al. (2025) conducted a retrospective study showing that ultrasound‐guided tip localization achieved a higher one‐time puncture success rate, lower incidence of venous leakage, pain reactions and complications (6.00% vs. 20.00%) compared to external measurement, confirming the superior clinical value of B‐ultrasound localization in MC tip positioning (Yu et al. 2025).

Elli et al. (2020) explored ultrasound‐guided tip location for MCs. The technique proved feasible in 98.9% of patients. They concluded the ideal tip site is the axillary vein, noting this ultrasound method is safe, low‐cost and practical. However, Thiyagarajanand & Ravindran demonstrated the feasibility of ultrasound‐guided catheter tip localization, yet successful confirmation that the catheter tip resided within the axillary vein was attained in merely 75% of patients (Thiyagarajanand and Ravindran 2022).

Despite its advantages, the ultrasound technique relies on subjective operator judgement for probe positioning and tip adjustment, lacking objective, quantifiable anatomical benchmarks for consistent placement.

#### 3.4.3 Plain Chest Radiography

Plain radiography has traditionally been a common confirmation method, with nine studies (24.39%) utilizing X‐ray. On chest X‐ray, the lateral border of the scapula is often cited as the anatomical boundary for the axillary vein. Therefore, an MC tip proximal to this border was considered to be in the axillary vein, while a tip distal to it was considered to be in the brachial vein (Schechter et al. 2024).

However, the routine application of chest X‐ray for MC confirmation is increasingly controversial due to several distinct limitations. First, chest X‐ray demonstrated limited visualization capability, failing to detect the catheter tip in 28.14% of cases (47 out of 167 patients) (Schechter et al. 2024). Second, routine radiographic confirmation for non‐central devices introduces an ethical tension regarding unnecessary radiation exposure, contravening the ALARA (As Low As Reasonably Achievable) principle. Third, chest X‐ray results require delayed reporting (awaiting radiologist acquisition and interpretation), which impedes real‐time clinical decision‐making and delays the initiation of intravenous therapy.

### 3.5 Factors Associated With Tip Positioning Accuracy

The accuracy of midline catheter (MC) tip placement is not determined by a single variable but is the result of a complex interplay of patient anatomy, device characteristics and operator technique. Understanding these factors is crucial for developing strategies to improve placement success and reduce complications.

#### 3.5.1 Obesity

In patients with a high body mass index (BMI), external anatomical landmarks become obscured, reducing the reliability of surface measurements. More critically, ultrasound visualization—the cornerstone of modern guidance—can be severely limited in cases of morbid obesity due to increased depth of target veins and poor sonographic penetration through adipose tissue. One study reported that in approximately 1.1% of patients (specifically those with morbid obesity), the MC tip could not be definitively identified via ultrasound, necessitating alternative confirmation methods or resulting in indeterminate placement (Elli et al. 2020).

#### 3.5.2 Insertion Site

The choice of puncture site directly dictates the catheter's intravascular path and final tip destination. While the mid‐upper arm (often referred to as Dawson's ‘green zone’) is a common starting point, its width introduces substantial variability (Huang et al. 2023). A puncture in the proximal versus distal end of this zone can alter the catheter's trajectory by several centimetres. Consequently, even when clinicians place fixed‐length midline catheters via the basilic vein uniformly, a difference of several centimetres in puncture height within the Green Zone may shift the catheter's terminal tip from the distal axillary vein all the way to the brachiocephalic vein. This highlights that ‘midclavicular’ positioning is not an anatomically precise term but a broad target zone heavily influenced by the initial puncture site (Elli et al. 2020).

#### 3.5.3 Catheter Length

The included studies reported MC lengths ranging from 15 to 40 cm, with 15–20 cm being the most commonly used range. Length selection must be tailored to the individual patient's anatomy and chosen insertion site. Reliance on a single ‘standard’ length without consideration of individual patient measurements is a key source of tip malposition, either too proximal (in small veins, increasing thrombosis risk) or too distal (unintentionally central).

#### 3.5.4 Catheter Tip Configuration

The design of the catheter tip influences both placement success and complication rates. A study utilizing a team catheter database showed that open‐tipped catheters triggered more complications and higher removal rates compared to closed‐tipped catheters (Zerla et al. 2015). The studies summarized in Table 2 included different catheter tip designs, including ‘open‐ended, non‐valved’ catheters (Passaro et al. 2025) and ‘three‐way valve‐type’ catheters (Xiong et al. 2023; Zeng et al. 2025). Valved or closed‐tip catheters are designed to limit blood reflux into the catheter lumen, thereby reducing the risks of intraluminal occlusion, thrombus formation and catheter dysfunction. However, because catheter tip design is often reported inconsistently across studies, its independent effect on MC‐related outcomes remains difficult to determine and warrants further investigation.

#### 3.5.5 Catheter‐To‐Vein Ratio (CVR) at the Tip Level

The proportion of venous lumen occupied by the distal catheter segment—directly modulates local blood flow and the risk of catheter‐related thrombosis. In their 2023 retrospective cohort study of midline and long peripheral catheters, Fabiani et al. identified that a tip‐level CVR exceeding 45% served as an independent strong predictor of composite catheter failure events such as symptomatic venous thrombosis (Fabiani et al. 2023). In a subsequent 2024 prospective HERITAGE cohort from the same research team, bivariate analysis further demonstrated that tip‐level CVR above 33% was significantly correlated with elevated catheter failure risk; the 33% threshold also aligns with clinical ultrasound screening standards for peripheral venous access (Fabiani et al. 2024). Collectively, these two distinct cutoffs from successive investigations highlight the necessity of matching catheter outer diameter to target vein calibre, particularly for small‐calibre vessels, to reduce thrombotic complications.

#### 3.5.6 Catheter Material

The effect of catheter material and design on MC‐related complications remains uncertain because device characteristics were inconsistently reported across the studies summarized in Table 2. Catheters are typically made from silicone or polyurethane. Polyurethane catheters were frequently used in European and North American studies, whereas silicone three‐way valve‐type catheters were commonly reported in Chinese studies. Although some modern MCs incorporate antithrombogenic or antimicrobial coatings, their clinical benefit is still unclear. For instance, the Bahl et al. (2021) trial compared a 4F antithrombotic MC (MC‐AT, with a bonded polymer) to a 4.5F antithrombotic and antimicrobial MC (MC‐AT‐AM, with a chlorhexidine coating) and found no statistically significant difference in catheter‐related thrombosis rates (7.5% vs. 11.3%, *p* = 0.46). These results suggest that the clinical benefit of these technologies in reducing thrombosis has not yet been demonstrated and highlight that material and coating modifications alone cannot fully address the problem of CRT. Because most studies have not directly compared different materials while controlling for confounding factors, the independent effect of catheter material on complications remains an unresolved issue.

#### 3.5.7 Catheter Dwell Time

The intended use of MCs is for intermediate‐term therapy, but actual dwell times vary considerably, as shown in Table 4. Report dwell times ranging from 5 to 12 days (Zeng et al. 2025) to more than 150 days (Liu et al. 2019). Longer dwell time may increase cumulative exposure to mechanical irritation, endothelial contact, biofilm formation, thrombosis and catheter dysfunction. However, short dwell time may also reflect early catheter failure or early removal due to complications. Therefore, dwell time should not be interpreted only as a marker of catheter success; it should be analysed together with catheter indication, therapy completion, premature removal and complication rates.

**TABLE 4:** Evidence map of reported catheter‐related complications by midline catheter tip position.

Complication outcome^a^	Arm vein	Axillary vein	Distal axillary vein	Proximal axillary vein	Subclavian vein%	Brachiocephalic vein
Incidence of overall catheter‐related complications	—	0.979%–40.00% (Nie et al. 2025; Xiong and Zhang 2025; Jiang et al. 2024; Wu et al. 2020; Lang et al. 2020; Gu et al. 2020; Bai and Guo 2019)	31.33% (Zhao et al. 2023)	4.76%–60.0% (Che et al. 2026; Zhu et al. 2025; Zhao et al. 2023; Yu et al. 2023; Xiong et al. 2023; Bai et al. 2022)	0.845%–25.00% (Yu et al. 2026; Chen et al. 2026; Che et al. 2026; Nie et al. 2025; Jiang et al. 2024; Zhao et al. 2023; Zhu et al. 2025; Yu et al. 2025; Yu et al. 2023; Zeng et al. 2023; Chen et al. 2023; Bai et al. 2022; Wu et al. 2020; Lang et al. 2020; Gu et al. 2020; Bai and Guo 2019; Liu and Li 2019)	17.5% (Liu et al. 2019)
Incidence of phlebitis	—	2.00%–15.0% (Jing 2023; Wu et al. 2020; Lang et al. 2020; Gu et al. 2020; Bai and Guo 2019; Liu and Li 2019)	0.83% (Zhao et al. 2022)	0.95%–15.0% (Yu et al. 2023; Xiong et al. 2023)	1.49%–5.33% (Mao 2023; Yu et al. 2023; Wu et al. 2020; Lang et al. 2020; Gu et al. 2020; Bai and Guo 2019; Liu and Li 2019)	1.25% (Liu et al. 2019)
Incidence of infiltration	—	1.67%–13.33% (Xiong and Zhang 2025; Jiang et al. 2024; Lang et al. 2020)	13.25%–14.17% (Zhao et al. 2023, 2022)	5.00%–7.87% (Che et al. 2026; Zhu et al. 2025; Zhao et al. 2023, 2022)	0.085%–7.87% (Che et al. 2026; Nie et al. 2025) 1.67%–4.35% (Lang et al. 2020)	—
Incidence of oozing	—	0.089%–13.33% (Nie et al. 2025; Xiong and Zhang 2025; Lang et al. 2020)	—	—	1.04%–4.35% (Zeng et al. 2023; Lang et al. 2020)	—
Incidence of bleeding	—	0.447%–5.97% (Nie et al. 2025; Gu et al. 2020)	13.25%–13.33% (Zhao et al. 2023, 2022)	0.95%–7.34% (Che et al. 2026; Zhu et al. 2025; Xiong et al. 2023; Zhao et al. 2023, 2022)	0.338%–7.50% (Che et al. 2026; Nie et al. 2025; Zhu et al. 2025; Yu et al. 2025; Chen et al. 2023; Zhao et al. 2023, 2022)	—
Incidence of catheter occlusion	—	2.50%–30.0% (Xiong and Zhang 2025; Mao 2023; Thiyagarajanand and Ravindran 2022; Wu et al. 2020; Lang et al. 2020; Bai and Guo 2019; Liu and Li 2019)	7.23%–10.83% (Zhao et al. 2023, 2022)	0.92%–30.0% (Che et al. 2026; Zhao et al. 2023; Zhu et al. 2025; Yu et al. 2023; Zhao et al. 2022; Bai et al. 2022)	0.254%–8.7% (Yu et al. 2026; Nie et al. 2025; Yu et al. 2025; Chen et al. 2023; Yu et al. 2023; Zhao et al. 2023; Zhao et al. 2022; Bai et al. 2022; Wu et al. 2020; Lang et al. 2020; Bai and Guo 2019; Liu and Li 2019)	2.5% (Liu et al. 2019)
Incidence of catheter dislodgement	—	0.356%–13.33% (Nie et al. 2025; Jiang et al. 2024; Mao 2023; Xiong and Zhang 2025; Bai and Guo 2019)	8.43%–9.17% (Zhao et al. 2023; Zhao et al. 2022)	1.05%–7.50% (Che et al. 2026; Zhao et al. 2023; Zhu et al. 2025; Xiong et al. 2023; Zhao et al. 2022)	0.169%–10% (Che et al. 2026; Nie et al. 2025; Zhu et al. 2025; Yu et al. 2025; Zeng et al. 2023; Chen et al. 2023; Zhao et al. 2023; Zhao et al. 2022; Bai and Guo 2019)	3.75% (Liu et al. 2019)
Incidence of catheter‐related infection	—	0.089%–7.14% (Nie et al. 2025; Jiang et al. 2024; Thiyagarajanand and Ravindran 2022; Liu and Li 2019)		—	1.25%–4.76% (Chen et al. 2026; Liu and Li 2019)	—
Incidence of catheter‐related thrombosis	41.5% (Schechter et al. 2024)	1.10%–38.9% (Jiang et al. 2024; Schechter et al. 2024; Thiyagarajanand and Ravindran 2022; Elli et al. 2020; Wu et al. 2020; Lang et al. 2020; Liu and Li 2019)	1.67%–2.41% (Zhao et al. 2023; Zhao et al. 2022)	0.81%–10.00% (Che et al. 2026; Zhu et al. 2025; Yu et al. 2023; Zhao et al. 2023; Bai et al. 2022; Zhao et al. 2022)	0.085%–3.33% (Chen et al. 2026; Nie et al. 2025; Jiang et al. 2024; Chen et al. 2023; Yu et al. 2023; Wu et al. 2020; Lang et al. 2020; 5.97% Gu et al. 2020; Bai and Guo 2019)	1.25% (Liu et al. 2019)
Unplanned catheter removal	—	0.49%–3.00% (Jing 2023; Gu et al. 2020)	—	0.95% (Xiong et al. 2023)	0.870%–6.00% (Yu et al. 2025; Zeng et al. 2023; Wu et al. 2020)	5.0% (Liu et al. 2019)
Incidence of malposition	—	0.089% (Nie et al. 2025)	—	—		2.5% (Liu et al. 2019)

Abbreviation: —, the corresponding complication was not reported for that tip position in the included studies.

^a^ The values in the table represent the range (minimum value–maximum value) of incidence rates reported across all supporting studies for a specific complication and tip location. A single percentage indicates that only one study reported this data. Complication rates were summarized descriptively rather than pooled statistically. The wide and unweighted ranges presented should not be interpreted as comparative effect estimates.

### 3.6 Tip Position and Clinical Complications

The position of the midline catheter tip may influence device performance and the risk of catheter‐related complications, although the strength and consistency of this association vary across studies. Understanding this relationship is crucial for optimizing patient safety and catheter dwell time. Table 4 provides a structured overview of common catheter‐related complications and their reported incidence across various MC tip locations as observed in the included primary studies.

It should be noted that some studies did not analyse complications according to standard anatomical tip locations. Fu et al. (2023), for example, compared a superficial implementation group with a deep implementation group and found a significantly higher complication rate in the superficial group than in the deep group (23.81% vs. 7.55%, *p* = 0.026) (Fu et al. 2023). Although this finding suggests that catheter depth or a more proximal/deeper tip position may be clinically relevant, this classification cannot be directly equated with anatomical categories such as the distal axillary vein, thoracic segment of the axillary vein, or subclavian vein.

Conversely, Passaro et al. (2025) found no statistically significant association between catheter tip position and fibroblastic sleeve (FS) formation or CRT. Among 27 enrolled patients, 10 developed FS and 2 developed symptomatic CRT, with no asymptomatic CRT observed. The incidence of FS formation was 38.10% versus 33.33%, and CRT was 9.52% versus 0% between the ‘correct’ and ‘incorrect’ tip‐position groups, respectively. Fisher's exact test showed no significant association between tip position and either FS formation or CRT, with *p* = 1.000 for both outcomes (Passaro et al. 2025).

## 4 Discussion

Midline catheters (MCs) have been increasingly used in clinical practice due to their longer indwelling time compared to short peripheral catheters and lower risk of central venous catheter‐related bloodstream infections compared to peripherally inserted central catheters (PICCs) (Yasuda et al. 2026). However, this scoping review reveals a core contradiction: although the tip position of the catheter is a key factor in determining its functionality, safety and risk of complications, there is currently no clarity.

No consistent, evidence‐based standard for defining the optimal tip location, confirming its position, or standardizing the factors that influence accuracy.

### 4.1 Inconsistent Definitions and Classification Standards

Our review found that target veins are described using a wide range of terms, including ‘axillary vein’, ‘arm vein’, ‘thoracic segment of the axillary vein’, ‘distal axillary vein’, ‘proximal axillary vein’ and ‘brachiocephalic vein’ (Passaro et al. 2025; Schechter et al. 2024; Yu et al. 2023; Zhao et al. 2023). In contrast, other research groups adopt precise anatomical localization methods, such as measuring the linear distance between the midline catheter tip and the clavicle via ultrasound imaging (Fu et al. 2023). This inconsistency reflects the historical evolution of guideline recommendations: earlier guidance emphasized external landmarks such as ‘at or near the level of the axilla’ (Gorski 2017), while more recent approaches have attempted to define anatomical boundaries using ‘thoracic tract of the axillary vein or subclavian vein’ (van Rens et al. 2025). No consensus has emerged, and the persistent ambiguity in terminology directly contributes to substantial between‐study heterogeneity and makes cross‐study comparisons extremely challenging.

### 4.2 Limitations of Tip Confirmation Methods

The choice of confirmation methods varies significantly and has its respective limitations.

The external body surface measurement method is the most widely used bedside method, and its advantages lie in its simplicity, speed and the absence of additional equipment. However, its accuracy heavily depends on the operator's experience, individual patient anatomical variations (such as obesity, edema and joint range of motion) (Zeng et al. 2023; Zeng et al. 2025), and the selected body surface landmarks. Essentially, it is an indirect estimation and cannot account for abnormal vessel courses or catheter malpositioning.

Ultrasound technology has become a key adjunctive tool for midline catheter (MC) insertion and post‐insertion assessment. Real‐time ultrasound guidance improves first‐attempt success and reduces early mechanical complications (Gilmore et al. 2025). For tip assessment, ultrasound can be used either by direct visualization of the catheter or indirectly through a saline‐flush technique that produces a transient ‘microbubble sign’ (Gidaro et al. 2023). However, conventional ultrasound assessment is often limited to the upper arm or axillary region and may not allow continuous visualization of the catheter course as it advances proximally (Annetta et al. 2026). Supraclavicular ultrasound can complement this approach by providing an acoustic window to the subclavian vein, brachiocephalic venous confluence, distal internal jugular vein and adjacent proximal venous structures (Yu et al. 2025). When a MC is advanced towards the thoracic segment of the axillary vein or the axillary–subclavian transition, supraclavicular ultrasound may help track catheter or guidewire progression and provide real‐time confirmation of the terminal tip location at or near the intended target zone. Despite these advantages, ultrasound assessment remains operator‐dependent and technically challenging, particularly in deep veins or complex anatomical regions. Therefore, ultrasound should be regarded as a valuable adjunct rather than a fully standardized standalone method.

Chest X‐ray radiographs have long been regarded as the reference standard for confirming the tip position of vascular access devices. In the studies covered in this review, only a quarter of them used it as a confirmation method. For midline catheters, its drawbacks are particularly prominent: (1) Insufficient sensitivity: One study reported that it failed to detect the catheter tip in up to 28.14% of cases (Schechter et al. 2024), and its imaging ability for non‐central veins is limited; (2) Radiation exposure: Although the single dose is low, there are ethical considerations for using X‐rays indiscriminately for multiple catheter insertions or imaging examinations in patients who may require multiple insertions or imaging; (3) Decision delay: Waiting for the radiologist's report makes it impossible to achieve immediate bedside confirmation and adjustment of the catheter, losing the opportunity for real‐time optimization.

### 4.3 Factors Associated With Tip Positioning Accuracy

Our review identified several factors that may influence the accuracy of MC tip positioning, including patient‐related factors (e.g., insertion site, body mass index, anatomical variation), device‐related factors (e.g., catheter material, length, tip configuration, dwell time) and operator‐related factors (e.g., experience, skill in ultrasound). However, magnitude of these effects was inconsistent across studies. For example, Zhao et al. (2023) classified midline catheter tips into subclavian vein, thoracic tract of the axillary vein and distal axillary vein groups, and reported catheter dwell times that were generally longer in the subclavian or thoracic axillary vein groups than in the more distal group. Similarly, Fu et al. grouped catheters according to the distance between the catheter tip and the clavicle and reported a longer median dwell time when the catheter tip was closer to the clavicle (Fu et al. 2023). The available studies differ significantly in catheter type, patient populations, insertion techniques, outcome definitions, and confirmation methods, making definitive conclusions challenging. Similarly, operator experience was cited as a key determinant in some studies, but the threshold for ‘experience’ was never standardized. This lack of consistent evidence further complicates the development of a universally applicable positioning protocol.

### 4.4 Tip Location and Clinical Complications

The relationship between tip location and clinical complications is the ultimate test of any positioning strategy. A key insight from this review is that an overemphasis on tip location alone may obscure the multifactorial nature of MC‐related complications. Our findings on catheter dwell time illustrate this point perfectly. While MCs are intended for intermediate‐term therapy, reported dwell times vary dramatically, from a median of 5–12 days (Zeng et al. 2025) to over 150 days (Liu et al. 2019). This variability, coupled with the uncertainty surrounding the impact of catheter materials (e.g., silicone vs. polyurethane), suggests that device failure and complications are the result of a complex interplay between patient factors, material biocompatibility, dwell duration and tip location. The inability of most studies to control for these confounding variables is a primary reason why the independent effect of tip position remains an unresolved question. Our review mapped a broad range of reported complications, including catheter‐related thrombosis, phlebitis, occlusion, catheter dysfunction, dislodgement and pain. Three recent meta‐analyses have attempted to quantify this relationship. Taken together, these meta‐analyses suggest a potential signal that more proximal tip locations—particularly the subclavian vein—may be associated with fewer complications. However, their findings are not fully consistent: Zhang et al. found significant differences for only overall complications and thrombosis (Zhang et al. 2024), whereas Sheng et al. and Yang et al. reported broader protective associations (Sheng et al. 2025; Yang et al. 2025). These inconsistencies likely reflect the same problems identified in this review: differing anatomical categories, variable confirmation methods and heterogeneous outcome definitions. Some studies compared the subclavian vein with the axillary vein without specifying whether the latter referred to the distal, proximal, thoracic, or chest‐wall segment, while others included transitional zones such as the axillo‐subclavian junction or brachiocephalic vein. Complications were also defined differently: phlebitis was not consistently classified as mechanical, chemical, or infectious; catheter‐related thrombosis was variously defined as symptomatic thrombosis, ultrasound‐detected thrombosis, or catheter‐associated thrombosis; and dwell time was reported using different summary statistics. Therefore, although the existing evidence collectively points towards a potential benefit of more proximal placement, this conclusion should be regarded as a signal, not a definitive placement standard. Because of the fundamental heterogeneity in definitions, methods and outcomes, no specific optimal MC tip location can yet be recommended.

### 4.5 Implications for Nursing Practice

The current non‐standardized state of practice reflects the transitional stage of this field from experience to science.

Future research and clinical practice could focus on:

1. Standardization: Through consensus of professional societies, define the operational definitions of ‘deep’ and ‘shallow’ implantation and different target veins based on anatomy and physiology. Standardized anatomical terminology should be adopted across future studies to enable meaningful cross‐study comparisons.
2. Process optimization: Establish a clinical operation pathway based on multimodal confirmation, limit unnecessary use of X‐rays, and improve catheter insertion efficiency and safety. Questions of educational design, skill acquisition and interprofessional collaboration between nurses and physicians will be central to translating future evidence into everyday clinical practice.
3. Evidence generation: Conduct prospective cohort studies or randomized controlled trials using precise positioning techniques to clarify the relationship between different tip positions and the incidence of complications, catheter lifespan and patient comfort.

### 4.6 Study Limitations and Uncertainties

1. Potential over‐restrictive exclusion of ‘Mini‐midlines’: The protocol defines Midline Catheters (MCs) as ‘longer than 15 cm’ and explicitly excludes ‘Mini‐midlines’ (< 15 cm). This strict cutoff may omit a key subset of intermediate‐length catheters, especially as the definition of ‘Midline’ varies across institutions, excluding relevant transitional evidence.
2. Language restriction: The inclusion criteria limit studies to those published in English and Chinese. While resource constraints are understandable, excluding publications in other major languages (e.g., German, French, Spanish) may introduce geographical/clinical bias, as practice patterns in vascular access may differ notably in European and Latin American settings.
3. Lack of formal quality appraisal: As a scoping review, the primary objective is to map evidence rather than critically appraise individual study quality. However, if the review aims to inform clinical recommendations, the absence of a quality assessment (e.g., via a risk of bias tool) limits insight into evidence reliability.
4. No attempt to obtain missing data: The authors did not contact study authors for unreported data, which is acceptable in a scoping review but may result in incomplete extraction of details such as complication rates or tip confirmation methods.
5. Geographic bias: 75% of the studies were from China, which restricts the generalizability of the conclusions. Accordingly, any international clinical guidance developed from this body of evidence must be interpreted cautiously with this regional overrepresentation acknowledged. Catheter specifications, insertion training protocols, and patient characteristics within this predominantly Chinese evidence base may not translate directly to healthcare systems in other regions.
6. A potential source of selection bias in this review is the exclusion of articles for which the full text was unavailable. Out of 133 articles assessed at the full‐text stage, we were unable to retrieve the full text for seven articles despite significant efforts, including institutional library access, inter‐library loan requests and direct searches via Google Scholar. Furthermore, 24 records were excluded because they were conference abstracts or study registrations without a corresponding full‐text publication. While the exclusion of these 31 records (23.3% of those assessed at full‐text) is a common challenge in scoping reviews, it may have led to the omission of relevant data, particularly emerging findings often presented in conference proceedings. The potential impact of this missing evidence on our conclusions is acknowledged.

## 5 Conclusion

This scoping review has mapped the evidence surrounding midline catheter tip location, confirmation methods and associated clinical outcomes. The findings demonstrate substantial variation in clinical practice. Tip‐confirmation practices were heterogeneous, ranging from external anatomical measurement to ultrasound‐guided placement and post‐procedural radiography. The relationship between tip location and catheter‐related complications appears to be multifactorial and may be influenced by catheter characteristics, duration of catheter use, patient anatomy and clinical condition. This scoping review identifies that current evidence is inadequate to support a clear preference for any single midline catheter tip‐positioning strategy. This review provides a clear agenda for future research to fill these gaps, which is essential for developing robust, evidence‐based guidelines to optimize the safety and effectiveness of midline catheters.

## Author Contributions

**Jieyu Wang:** methodology, formal analysis, supervision, writing – review and editing, project administration, conceptualization. **Si Deng:** methodology, data curation, validation, formal analysis, writing – original draft, software, visualization. **Hui Hu:** writing – original draft, methodology, data curation, software, validation, formal analysis, visualization. **Ya Feng:** data curation, formal analysis, writing – review and editing. **Xiaoling Dong:** data curation, formal analysis, writing – review and editing. **Jing Zhang:** data curation, formal analysis, writing – review and editing. **Jiao Yang:** data curation, formal analysis, writing – review and editing. **Li Geng:** methodology, formal analysis, writing – review and editing, supervision, conceptualization. **Yilan Liu:** supervision, methodology, formal analysis, writing – review and editing, conceptualization. **Yaling Wang:** methodology, formal analysis, supervision, writing – review and editing, project administration, conceptualization.

## Funding

The authors have nothing to report.

## Ethics Statement

Ethics approval was not required for this review because only published literature was used.

## Conflicts of Interest

The authors declare no conflicts of interest.

## Supporting information


**Table S1:** Search strategies used in data sources.

**Table S2:** Characteristics of Included Publications.

## Data Availability

The data that support the findings of this study are available from the corresponding author upon reasonable request.
