# Supplementary material for: Mapping the Evidence on Midline Catheter Tip Location, Confirmation Methods and Associated Characteristics: A Scoping Review
Source: Nurs Open. 2026 Sep 30;13(10):e70876. doi: 10.1002/nop2.70876 (PMC13625669; doi:10.1002/nop2.70876)
Supplement: Supplementary file 1 [file NOP2-13-e70876-s001.docx]

Table 1. Search strategies used in data sources.

| Databases | Search query |
| --- | --- |
| Cochrane Library | ("midline catheter*" OR "mid-line catheter*" OR "midline venous catheter*" OR "midline peripheral catheter*" OR "medium-term intravenous access*" OR "medium-long catheter*" OR "midlength catheter*" OR "mid-length catheter*" OR "medium and long catheter*" OR "midclavicular catheter*" OR "long peripheral catheter*" OR "long peripheral cannula" OR "extended dwell catheter") AND ("tip" OR "position" OR "positioning" OR "location" OR "subclavian" OR "axillary" OR "brachiocephalic" OR "orientation") |
| PubMed | ("midline catheter*"[Title/Abstract] OR "mid-line catheter*"[Title/Abstract] OR "midline venous catheter*"[Title/Abstract] OR "midline peripheral catheter*"[Title/Abstract] OR "medium-term intravenous access*"[Title/Abstract] OR "medium-long catheter*"[Title/Abstract] OR "midlength catheter*"[Title/Abstract] OR "mid-length catheter*"[Title/Abstract] OR "medium and long catheter*"[Title/Abstract] OR "midclavicular catheter*"[Title/Abstract] OR "long peripheral catheter*"[Title/Abstract] OR "long peripheral cannula"[Title/Abstract] OR "extended dwell catheter"[Title/Abstract]) AND ("tip"[Title/Abstract] OR "position"[Title/Abstract] OR "positioning"[Title/Abstract] OR "location"[Title/Abstract] OR "subclavian"[Title/Abstract] OR "axillary"[Title/Abstract] OR "brachiocephalic"[Title/Abstract] OR "orientation"[Title/Abstract]) |
| EMBASE | ('midline catheter*':ti,ab,kw OR 'mid-line catheter*':ti,ab,kw OR 'midline venous catheter*':ti,ab,kw OR 'midline peripheral catheter*':ti,ab,kw OR 'medium-term intravenous access*':ti,ab,kw OR 'medium-long catheter*':ti,ab,kw OR 'midlength catheter*':ti,ab,kw OR 'mid-length catheter*':ti,ab,kw OR (medium:ti,ab,kw AND 'long catheter*':ti,ab,kw) OR 'midclavicular catheter*':ti,ab,kw OR 'long peripheral catheter*':ti,ab,kw OR 'long peripheral cannula':ti,ab,kw OR 'extended dwell catheter':ti,ab,kw) AND (tip:ti,ab,kw OR position:ti,ab,kw OR positioning:ti,ab,kw OR location:ti,ab,kw OR subclavian:ti,ab,kw OR axillary:ti,ab,kw OR brachiocephalic:ti,ab,kw OR orientation:ti,ab,kw) |
| Web of Science | **"midline catheter*" or "mid-line catheter*" or "midline venous catheter*" or "midline peripheral catheter*" or "medium-term intravenous access*" or "medium-long catheter*" or "midlength catheter*" or "mid-length catheter*" or "medium and long catheter*" or "midclavicular catheter*" or "long peripheral catheter*" or "long peripheral cannula" or "extended dwell catheter"** (Topic) and **"tip"or"position" or "positioning" or "location" or "subclavian" or "axillary" or "brachiocephalic" or "orientation"** (Topic) |
| Scopus | TITLE-ABS-KEY ( ( "midline catheter*" OR "mid-line catheter*" OR "midline venous catheter*" OR "midline peripheral catheter*" OR "medium-term intravenous access*" OR "medium-long catheter*" OR "midlength catheter*" OR "mid-length catheter*" OR "medium and long catheter*" OR "midclavicular catheter*" OR "long peripheral catheter*" OR "long peripheral cannula" OR "extended dwell catheter" ) AND ( "tip" OR "position" OR "positioning" OR "location" OR "subclavian" OR "axillary" OR "brachiocephalic" OR "orientation" ) ) |
| ProQuest | abstract("midline catheter*" OR "mid-line catheter*" OR "midline venous catheter*" OR "midline peripheral catheter*" OR "medium-term intravenous access*" OR "medium-long catheter*" OR "midlength catheter*" OR "mid-length catheter*" OR "medium and long catheter*" OR "midclavicular catheter*" OR "long peripheral catheter*" OR "long peripheral cannula" OR "extended dwell catheter") AND abstract("tip" OR "position" OR "positioning" OR "location" OR "subclavian " OR "axillary" OR "brachiocephalic" OR "orientation") |
| CINAHL | (XB ("midline catheter*") OR XB ("mid-line catheter*") OR XB ("midline venous catheter*") OR XB ("midline peripheral catheter*") OR XB ("medium-term intravenous access*") OR XB ("medium-long catheter*") OR XB ("midlength catheter*") OR XB ("mid-length catheter*") OR XB ("medium and long catheter*") OR XB ("midclavicular catheter*") OR XB ("long peripheral catheter*") OR XB ("long peripheral cannula") OR XB ("extended dwell catheter")) AND (XB ("tip") OR XB ("position") OR XB ("positioning") OR XB ("location") OR XB ("subclavian") OR XB ("axillary") OR XB ("brachiocephalic") OR XB ("orientation")) |
| CNKE | AB=(中线导管+中等长度静脉导管+中长度导管+中等长度导管+中长静脉导管+中长导管) AND AB=(尖端+头端+位置+定位) |
| Wangfang Data | 摘要: ("中线导管" or "中等长度静脉导管" or "中长度导管" or "中等长度导管" or "中长静脉导管" or "中长导管") and 摘要: ("尖端" or "头端" or "位置" or "定位") |
| Sinomed | ("中线导管"[摘要:智能] OR "中等长度静脉导管"[摘要:智能] OR "中长度导管"[摘要:智能] OR "中等长度导管"[摘要:智能] OR "中长静脉导管"[摘要:智能] OR "中长导管"[摘要:智能]) AND ("尖端"[摘要:智能] OR "头端"[摘要:智能] OR "位置"[摘要:智能] OR "定位"[摘要:智能]) |
| VIP database | R=(中线导管 OR 中等长度静脉导管 OR 中长度导管 OR 中等长度导管 OR 中长静脉导管 OR 中长导管) AND R=(尖端 OR 头端 OR 位置 OR 定位) |
