# Supplementary material for: Mapping the Evidence on Midline Catheter Tip Location, Confirmation Methods and Associated Characteristics: A Scoping Review
Source: Nurs Open. 2026 Sep 30;13(10):e70876. doi: 10.1002/nop2.70876 (PMC13625669; doi:10.1002/nop2.70876)
Supplement: Supplementary file 2 [file NOP2-13-e70876-s002.docx]

Table S2. Characteristics of Included Publications

| Title of article | Authors, year | Countries | Study design^*^ | Midline-specific sample size (n) |
| --- | --- | --- | --- | --- |
| The application of medium-length catheters in the infusion of drugs with extreme pH values in patients with severe HDU | M. Yu et al., 2026 | China | RCT | Patients with severe HDU who are hospitalized and require intravenous infusion of extreme pH value drugs (43) |
| The application of different types of venous vascular access in special medications for patients in the Intensive Care Unit | Z. Chen et al., 2026 | China | RCT | Patients in the Intensive Care unit who need to be infused with blistering agents, extreme pH value drugs, and high osmotic pressure drugs (80) |
| The impact of different external measurement methods combined with ultrasound-guided technique for inserting mid-length peripheral intravenous catheters in hospitalized patients on hospitalized patients ^a^ | Che et al., 2026 | China | NRCT | Inpatient (191) |
| A Clinical Randomized Controlled Trial of Complication Rates with Four Different Tip Positions for FrontOpening Midline Catheters ^a^ | Che, 2025 | China | RCT | Inpatient (476) |
| Nursing care for midline catheter insertion in 12 patients with severe obesity | X. Zeng et al., 2025 | China | Case study | Patients with severe obesity (12) |
| Incidence of fibroblastic sleeve in midline catheters: A pilot study in a non-intensive medical department | Passaro et al., 2025 | Italy | Cohort study | Patients admitted to the Internal Medicine Wards (41) |
| Impact of silicone midline catheter tip location on catheter-related complications and indwelling duration: Results from a multicentre randomised clinical trial in China | Nie et al., 2025 | China | RCT | Patients underwent MC placement (2307) |
| Comparison of catheter-related complications, indwelling time and extubation rate due to complications at different tip positions of medium-length venous catheters | Zhu et al., 2025 | China | RCT | Inpatients (80) |
| Application of B-Ultrasound Localization in Midline Catheterization | L. Yu et al., 2025 | China | Cohort study | Oncology patients (100) |
| Discussion on the application value of modified medium and long catheters in cancer patients in primary hospitals | F. Xiong & Zhang, 2025 | China | RCT | Oncology patients (30) |
| Midline catheter-associated thrombosis (MCAT): Does tip location in the axillary vein increase risk? | Schechter et al., 2024 | USA | Cohort study | Hospitalized patients who not only had a midline catheter but also had a chest x-ray and venous duplex performed (59) |
| The longer the catheter, the lower the risk of complications: Results of the HERITAGE study comparing long peripheral and midline catheters | Fabiani et al., 2024 | Italy | Cohort study | Cardiac Surgery, Cardiology, and Internal Medicine Departments of hospitalized patients（131） |
| The influence of placing the tip of a medium-length catheter at different positions on the incidence of complications | Jiang et al., 2024 | China | Cohort study | Inpatients (184) |
| Midline catheter tip position and catheter-related complications in antimicrobial therapy: A multi-center randomized controlled trial ^b^ | Zhao et al., 2023 | China | RCT | Patients estimated time of intravenous infusion ≥1 week before catheterization in six first-class general hospitals in China（281） |
| Midline catheters for extracorporeal photopheresis in hematological patients | Marche et al., 2023 | Italy | Descriptive study | All consecutive adult patients (≥18 years old) with a diagnosis of chronic GVHD and candidate to ECP treatment (69) |
| Contrast enhanced ultrasound as a new tool to estimate the performance of midline catheters in the single patient | Gidaro et al., 2023 | Italy | Cohort study | Patients who had a difficult intravenous access (DIVA) and an expected need of intravenous therapy longer than 6 days (132) |
| Impact of terminal tip location of midline catheters on catheter-related complications and indwelling duration in elderly patients | Fu et al., 2023 | China | Cohort study | Elderly patients (95) |
| The catheter-to-vein ratio at the tip level, not the catheter type, as a risk factor for a catheter failure. A retrospective comparative study of polyurethane midline and long peripheral cathetersT | Fabiani et al., 2023 | Italy | Cohort study | Adult patients (126) |
| Discussion on the application effect of different tip positions of the modified medium-length catheter | Y. Yu et al., 2023 | China | Cohort study | Inpatients (120) |
| Prospective clinical study on the safety of medium-length intravenous catheter infusion of parenteral nutrition solution | W. Xiong et al., 2023 | China | Self‑controlled study | Patients required intravenous infusion of parenteral nutrition solution for support (105) |
| safety comparison of midline catheter tip location in the anterior axillary line and midclavicular line | Mao, 2023 | China | Cohort study | Inpatients (150) |
| exploring the clinical effects of different infusion tools in orthopedic patients with Aescin sodium infusion | Jing, 2023 | China | RCT | Patients who receive Aescin sodium in orthopedics (100) |
| One-needle subcutaneous tunnel puncture in modified three-way valve medium and long catheters | X. Chen et al., 2023 | China | NRCT | Neurosurgical critically ill patients (80) |
| Comparison of the effect of different positions combined with modified skin expanion in midline catheter placement in patients with consciousness disorders | L. Zeng et al., 2023 | China | RCT | Patients with impaired consciousness (216) |
| Conventional central venouscatheters as tunnelled mid-clavicularmidline catheters: Description of novelapplication and outcome analysis | Thiyagarajanand & Ravindran, 2022 | India | Descriptive study | Patients aged 18 years and older who were referred to the anaesthesiology department for intermediate venous access (72) |
| Application comparison of different tip positions of midline catheters ^b^ | Zhao et al., 2022 | China | RCT | Inpatients (384) |
| Observation of the application of modified midline catheter tip position in continuous infusion of stimulating drugs in geriatrics | L. Yang et al., 2022 | China | Descriptive study | Inpatients with Infusion of stimulant drugs (100) |
| Effects of different tip positions of midline catheter in elderly patients | Bai et al., 2022 | China | Cohort study | Elderly patients (86) |
| Influencing factors of catheter⁃related complications in patients with intracranial hypertension treated with mannitol injection via midline catheter | Jin et al., 2021 | China | Descriptive study | Patients with high cranial pressure infused with mannitol injection (909) |
| Study on the application of modified medium-length catheters in infectious surgical hospitalized patients | M. Chen, 2021 | China | Descriptive study | Infected surgical inpatients (203) |
| Ultrasound-guided tip location of midline catheters | Elli et al., 2020 | Italy | Historical Control Study | Hospital patients (981) |
| Application effect of two lengths of three-way valve type medium and long catheters | W. Yang et al., 2020 | China | Cohort study | Inpatients (268) |
| Relationship between tip position of medium length catheter and complications | Wu et al., 2020 | China | Descriptive study | Neurology patients (111) |
| Observation of the application of modified medium-length catheters in neurosurgery | T. Wang et al., 2020 | China | Descriptive study | Neurosurgery patients (126) |
| Application of different midline catheters tip location in patients with hepatobiliary surgery related diseases | Lang et al., 2020 | China | Cohort study | Patients with diseases related to hepatobiliary surgery (120) |
| Correlation analysis of the intermediate and long catheter placement and indwelling time and the occurrence of complications in intravenous fluid therapy under ultrasound guidance | Gu et al., 2020 | China | Cohort study | Inpatients (134) |
| Clinical Application Effect of Ultrasound-guided Implant Modified Mid-length Catheter of Peripheral Venous with Modified Seldinger Technique | Z. Liu et al., 2019 | China | RCT | Inpatients (80) |
| Comparative study of midline catheters with different tip positions in the perioperative period of patients with gastrointestinal tumors | Y. Liu & Li, 2019 | China | RCT | Patients with gastrointestinal tumors (84) |
| Comparison of Midline Catheter Tip Positions Between the Anterior Axillary Line and the Midclavicular Line | Bai & Guo, 2019 | China | Cohort study | Elderly patients (80) |
| Modified length of medium length catheter insertion of peripheral veins Observation in neurology patients | Q. Wang et al., 2016 | China | Descriptive study | Neurology inpatients (185) |
| Open- vs closed-tip valved peripherally inserted central catheters and midlines: Findings from a vascular access database | Zerla et al., 2015 | Italy | Descriptive study | Adult oncology patients (623) |

^*^: RCT: Randomized Controlled Trial; NRCT: Non-Randomized Controlled Trial; non-RCT

^a^: Che et al. (2026) and Che, (2025) were approved by the same ethical review committee with the same approval ID (2023075, North China University of Science and Technology), and were identified as companion publications. Che et al. (2026) was designed as a non-randomized controlled trial (NRCT), whereas Che (2025, Master’s thesis) was a randomized controlled trial (RCT).

^b^: Zhao et al. (2022) and Zhao et al. (2023) were identified as companion publications originating from the same clinical trial (Registration No. NCT04601597).

**References**

Bai, J., & Guo, Y. (2019). Comparison of Midline Catheter Tip Positions Between the Anterior Axillary Line and the Midclavicular Line. *Electronic Journal of General Stomatology*, *6*(34), 133, 137. https://doi.org/DOI:10.16269/j.cnki.cn11-9337/r.2019.34.105

Bai, J., Guo, Y., Mo, Y., Wang, J., & Song, J. (2022). Effects of different tip positions of midline catheter in elderly patients. *Practical Geriatrics*, *36*(12), 1300～1303. https://doi.org/10.3969/j.issn.1003-9198.2022.12.028

Che, Y. (2025). *A Clinical Randomized Controlled Trial of Complication Rates with Four Different Tip Positions for FrontOpening Midline Catheters* [Master, North China University of Science and Technology]. https://doi.org/10.27108/d.cnki.ghelu.2025.000405

Che, Y., Liu, Z., Lu, Z., Ma, Y., & Jiao, G. (2026). The impact of different external measurement methods combined with ultrasound-guided technique for inserting mid-length peripheral intravenous catheters in hospitalized patients on hospitalized patients. *Nursing Practice and Research*, *23*(5), 777～783. https://doi.org/10.3969/j.issn.1672-9676.2026.05.021

Chen, M. (2021). Study on the application of modified medium-length catheters in infectious surgical hospitalized patients. *Modern Nurse*, *28*(4), 86～89. https://doi.org/10.19793/j.cnki.1006-6411.2021.12.029

Chen, X., Chen, X., Wei, C., Liu, Y., & Li, J. (2023). One-needle subcutaneous tunnel puncture in modified three-way valve medium and long catheters. *Nursing Practice and Research*, *20*(2), 291～293. https://doi.org/10.3969/j.issn.1672-9676.2023.02.027

Chen, Z., Li, M., Yu, M., Teng, W., & Li, J. (2026). The application of different types of venous vascular access in special medications for patients in the Intensive Care Unit. *Heilongjiang Medicine and Parmacy*, *49*(3), 126～128.

Elli, S., Pittiruti, M., Pigozzo, V., Cannizzo, L., Giannini, L., Siligato, A., Rondelli, E., Foti, G., & Lucchini, A. (2020). Ultrasound-guided tip location of midline catheters. *The Journal of Vascular Access*, *21*(5), 764～768. https://doi.org/10.1177/1129729820907250

Fabiani A., Aversana N., Santoro M., Calandrino D., Liotta P., & Sanson G. (2024). The longer the catheter, the lower the risk of complications: Results of the HERITAGE study comparing long peripheral and midline catheters. *American Journal of Infection Control*, *52*(11), 1289～1295. https://doi.org/10.1016/j.ajic.2024.06.019

Fabiani, A., Santoro, M., & Sanson, G. (2023). The catheter-to-vein ratio at the tip level, not the catheter type, as a risk factor for a catheter failure. A retrospective comparative study of polyurethane midline and long peripheral catheters. *Heart & Lung*, *60*, 39～44. https://doi.org/10.1016/j.hrtlng.2023.02.027

Fu, J., Zhao, L., Tian, Y., Liu, Y., Zhang, H., & Li, H. (2023). Impact of terminal tip location of midline catheters on catheter-related complications and indwelling duration in elderly patients. *Nursing Open*, *10*(4), 2349～2356. (162402681). https://doi.org/10.1002/nop2.1490

Gidaro, A., Casella, F., Lugli, F., Cogliati, C., Calloni, M., Samartin, F., Brena, N., & Pace, G. (2023). Contrast enhanced ultrasound as a new tool to estimate the performance of midline catheters in the single patient. *The Journal of Vascular Access*, *24*(2), 284～288. https://doi.org/10.1177/11297298211034629

Gu, H., Li, G., Fang, W., & Wang, L. (2020). Correlation Analysis of Catheter Tip Location, Dwell Time, and Complications of Midline Catheters under Ultrasound Guidance in Intravenous Therapy. *Today Nurse*, *27*(20), 144～146. https://doi.org/10.19791/j.cnki.1006-6411.2020.22.064

Jiang, F., Huang, T., & Chen, Y. (2024). The influence of placing the tip of a medium-length catheter at different positions on the incidence of complications. *Chinese Science and Technology Journal Database (Abstract Edition) - Medicine and Health*, (12), 115～118.

Jin, G., Ma, L., Wang, D., Tian, L., Wang, L., & Li, Y. (2021). Influencing factors of catheter⁃related complications in patients with intracranial hypertension treated with mannitol injection via midline catheter. *Journal of Xinxiang Medical University*, *38*(10), 941～943. https://doi.org/10.7683/xxyxyxb.2021.10.008

Jing, Q. (2023). Exploring the clinical effects of different infusion tools in orthopedic patients with Aescin sodium infusion. *Chinese Journal of Modern Medicine*, *25*(3), 93～95. https://doi.org/10.3969/j.issn.1672-9463.2023.03.024

Lang, L., Lu, H., Xiao, Q., & Zhang, Y. (2020). Application of different midline catheters tip location in patients with hepatobiliary surgery related diseases. *Clinical Medical Research and Practice*, *5*(30), 149～151. https://doi.org/10.19347/j.cnki.2096-1413.202030057

Liu, Y., & Li, C. (2019). Comparative study of midline catheters with different tip positions in the perioperative period of patients with gastrointestinal tumors. *Chinese Journal of Cancer Prevention and Treatment*, *26*(12), 211,213.

Liu, Z., Song, X., Kong, L., LIU, S., Luo, Z., & Yu, Y. (2019). Clinical Application Effect of Ultrasound-guided Implant Modified Mid-length Catheter of Peripheral Venous with Modified Seldinger Technique. *Chinese and foreign medical research*, *17*(35), 173～175. https://doi.org/10.14033/j.cnki.cfmr.2019.35.072

Mao, Y. (2023). Safety comparison of midline catheter tip location in the anterior axillary line and midclavicular line. *Journal of minimally invasive medicine*, *18*(1), 73～76. https://doi.org/10.11864/j.issn.1673.2023.01.17

Marche, B., D’Arrigo, S., Annetta, M. G., Musarò, A., Emoli, A., Sica, S., Piccirillo, N., Putzulu, R., De Paolis, M., Bernoldi, M., & Pittiruti, M. (2023). Midline catheters for extracorporeal photopheresis in hematological patients. *Journal of Vascular Access*, *24*(4), 568～574. https://doi.org/10.1177/11297298211041450

Nie, S., Wang, L., Ma, S., & Sun, H. (2025). Impact of silicone midline catheter tip location on catheter-related complications and indwelling duration: Results from a multicentre randomised clinical trial in China. *Bmj Open*, *15*(3), e086717. https://doi.org/10.1136/bmjopen-2024-086717

Passaro, G., Ortolani, E., Landolfi, E., Puzio, G., Zazzara, M. B., & Imperoli, G. (2025). Incidence of fibroblastic sleeve in midline catheters: A pilot study in a non-intensive medical department. *The Journal of Vascular Access*, *27*(2), 600～606. https://doi.org/10.1177/11297298251356774

Schechter, M. S., Baron, S. W., Gohari, A., Southern, W. N., & Galen, B. T. (2024). Midline catheter-associated thrombosis (MCAT): Does tip location in the axillary vein increase risk? *Journal of Infusion Nursing*, *47*(6), 363～368. Embase (181008599). https://doi.org/10.1097/NAN.0000000000000558

Thiyagarajanand, S., & Ravindran, C. (2022). Conventional central venouscatheters as tunnelled mid-clavicularmidline catheters: Description of novelapplication and outcome analysis. *The Journal of Vascular Access*, *23*(1), 98～104. https://doi.org/10.1177/1129729820982870

Wang, Q., Fu, R., Sun, J., & Shen, X. (2016). Modified length of medium length catheter insertion of peripheral veins Observation in neurology patients. *Journal of Nursing(China)*, *23*(15), 72～75. https://doi.org/10.16460/j.issn1008-9969.2016.15.072

Wu, H., Qiu, Y., & Chen, L. (2020). Relationship between tip position of medium length catheter and complications. *Lingnan Journal of Emergency Medicine*, *25*(5), 550～551. https://doi.org/10.3969/j.issn.1671⁃301X.2020.05.042

Xiong, F., & Zhang, H. (2025). Discussion on the application value of modified medium and long catheters in cancer patients in primary hospitals. *Contemporary Medical Theory Series*, *235*, 153～155. https://doi.org/0.3969/j.issn.2095-7629.2025.05.045

Xiong, W., Chen, J., Hong, W., Zhang, L., & Zhang, C. (2023). Prospective clinical study on the safety of medium-length intravenous catheter infusion of parenteral nutrition solution. *Chinese Journal of Modern Nursing*, *29*(5), 589～593. https://doi.org/10.3760/cma.j.cn115682-20220222-00812

Yang, L., Zhao, H., Miao, H., & Zhou, P. (2022). Observation of the application of modified midline catheter tip position in continuous infusion of stimulating drugs in geriatrics. *Healthful Friend*, (5), 127,129.

Yang, W., Chen, S., Zhong, Y., & Huang, Y. (2020). Application effect of two lengths of three-way valve type medium and long catheters. *Nursing Practice and Research*, *17*(11), 141～143. https://doi.org/10.3969/j.issn.1672-9676.2020.11.053

Yu, L., Li, Y., & Zhang, X. (2025). Application of B-Ultrasound Localization in Midline Catheterization. *China Medical Device Information*, *31*(3), 108～110. https://doi.org/10.15971/j.cnki.cmdi.2025.03.017

Yu, M., Chen, Z., Teng, W., & Li, M. (2026). The application of medium-length catheters in the infusion of drugs with extreme pH values in patients with severe HDU. *Heilongjiang Medicine and Parmacy*, *49*(3), 48～50.

Yu, Y., Liu, Z., Zheng, Y., Zhang, C., & Wang, X. (2023). Discussion on the application effect of different tip positions of the modified medium-length catheter. *International Journal of Nursing Research*, *5*(5), 50～52. https://doi.org/10.12208/j.ijnr.20230216

Zeng, L., Bao, S., Xiao, L., Yang, X., Liu, Y., & Lin, H. (2023). Comparison of the effect of different positions combined with modified skin expanion in midline catheter placement in patients with consciousness disorders. *Jilin Medicine*, *44*(7), 1954～1957.

Zeng, X., Cao, X., Chen, H., Wu, S., Jin, X., & Zhao, L. (2025). Nursing care for midline catheter insertion in 12 patients with severe obesity. *Chinese Journal of Nursing*, *60*(13), 1563～1566. https://doi.org/10.3761/j.issn.0254-1769.2025.13.005

Zerla, P. A., Canelli, A., Caravella, G., Gilardini, A., De Luca, G., Parini, R., & Gianoli, M. (2015). Open- vs closed-tip valved peripherally inserted central catheters and midlines: Findings from a vascular access database. *Journal of The Association for Vascular Access*, *20*(3), 169～176. https://doi.org/10.1016/j.java.2015.06.001

Zhao, L., Cai, Z., Fan, X., Jiang, F., Zhao, L., & Zhao, R. (2022). Application comparison of different tip positions of midline catheters. *Chinese Journal of Nursing*, *57*(5), 517～524. https://doi.org/10.3761/j.issn.0254-1769.2022.05.001

Zhao, L., Fan, X., Zhao, L., Cai, Z., Jiang, F., & Zhao, R. (2023). Midline catheter tip position and catheter-related complications in antimicrobial therapy: A multi-center randomized controlled trial. *International Journal of Nursing Studies*, *141*, 104476. https://doi.org/10.1016/j.ijnurstu.2023.104476

Zhu, H., Wang, J., & Bai, Y. (2025). Comparison of catheter-related complications, indwelling time and extubation rate due to complications at different tip positions of medium-length venous catheters. *Maternal and Child Care*, *5*(14), 3407～3409.
